# Crystal structure determination of *rac*-11′-(1-acetyl-1*H*-indazol-3-yl)-11′,11*a*′-di­hydro-10′*H*,17′*H*-spiro­[indene-2,18′-[5a,16*b*]methano­tri­indeno[1,2-*b*:1′,2′-*d*:2′′,1′′-*g*]oxocine]-1,3,10′,12′,17′(10*a*′*H*)-penta­one aceto­nitrile 1.5-solvate

**DOI:** 10.1107/S2056989018011763

**Published:** 2018-09-18

**Authors:** Mark Baranov, Radion Vainer, Mark V. Sigalov

**Affiliations:** a Department of Chemistry, Ben Gurion University of the Negev, POB 653, 8410501, Beer Sheva, Israel

**Keywords:** crystal structure, indan-1,3-dione, cyclization, condensation, eight-membered ring

## Abstract

The title compound, C_46_H_26_N_2_O_7_·1.5CH_3_CN, is the aldol condensation product of bindone with indazole-3-carbaldehyde followed by double inter­molecular cyclization. The asymmetric unit, which has monoclinic *P*2_1_/*c* symmetry, contains two independent mol­ecules of the title compound and three aceto­nitrile mol­ecules. The title mol­ecule comprises a central eight-membered ring, which contains an enol–ester, from which five arms extend. The arms exhibit inter­molecular inter­actions within the crystal lattice between mol­ecules of the title compound and with co-crystallized solvent mol­ecules (aceto­nitrile).

## Abstract 2

The title mol­ecule contains a central eight-membered ring, which contains an enol–ester, from which five arms extend. The arms exhibit inter­molecular inter­actions within the crystal lattice between mol­ecules of the title compound and with co-crystallized aceto­nitrile solvent mol­ecules.

## Chemical context   

1,3-Indandione derivatives have been known for more than a century and have found numerous applications as drugs (anti­coagulants, analgesics, anti-inflammatory medicines; Eriks *et al.*,1979 ▸), reagents in analytical and forensic chemistry (ninhydrins; Hansen & Joullié, 2005 ▸), dyes and pigments (Manukian & Mangini, 1970 ▸; Schelz, 1975 ▸; Bello *et al.*, 1987 ▸), semiconductors and photo semiconductors (Silinsh & Taure, 1969 ▸), and components of advanced materials (Gvishi *et al.*, 2003 ▸; Acharya *et al.*, 2005 ▸; Lokshin *et al.*, 2017 ▸). One of the important features of 1,3-indandione as well as its dimer bindone [2-(2,3-di­hydro-3-oxo-1*H*-inden-1-yl­idene)-1*H*-indene-1, 3(2*H*)-dione] is the ease of their self-condensation, often with the formation of complex cyclic structures (Jacob *et al.*, 2000 ▸). For over a century, cyclic 1,3-diketones have been known to form condensation products, including self-condensation (Wislicenus, 1887 ▸). As a result of this property, they have found use as inter­mediates for condensed cyclization products (Sekhar, 2004 ▸; Kozlov & Gusak, 2006 ▸) that have themselves found use as anti­emetic (Kuang *et al.*, 1994 ▸) and anti­cancer (Heidelberger & Ansfield, 1963 ▸) drugs. 
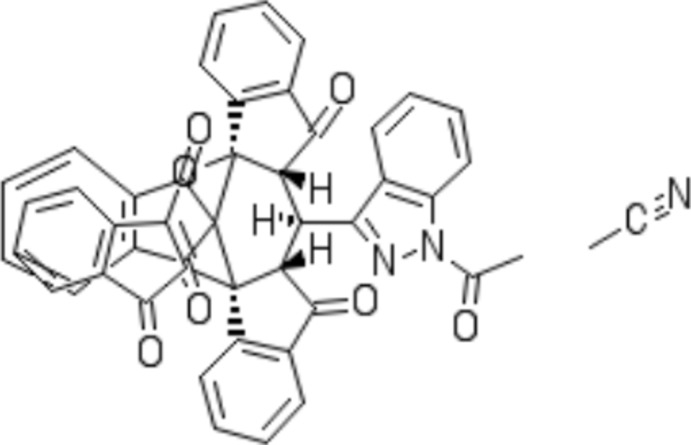


## Structural commentary   

**Figure 1:**
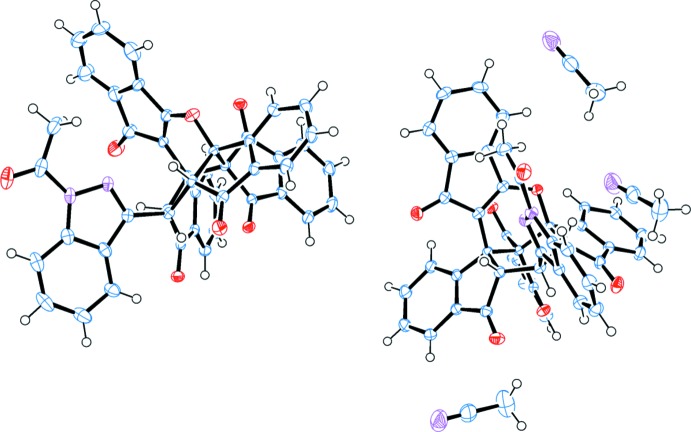
*ORTEP* representation of the asymmetric unit of the crystal, containing two compound mol­ecules and three co-crystallized aceto­nitrile mol­ecules. Displacement ellipsoids are drawn at the 50% probability level.

**Figure 2:**
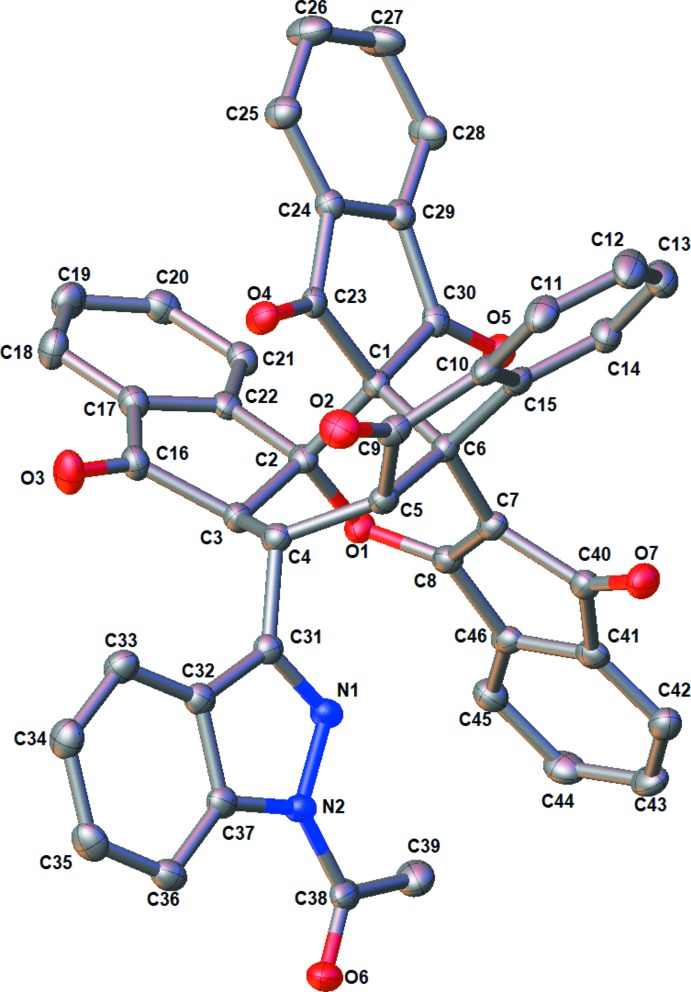
View of one of the independent mol­ecules in the title compound showing the atom-labelling scheme. Displacement ellipsoids are drawn at the 50% probability level.

The asymmetric unit of the title compound, shown in Fig. 1 ▸, contains two independent mol­ecules and three co-crystallized aceto­nitrile mol­ecules (*Z* = 8, *Z′* = 4). The title compound is shown in Fig. 2 ▸.

**Figure 3:**
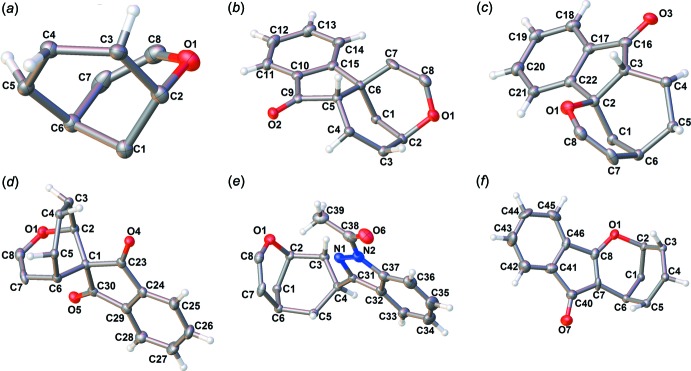
(*a*) The central eight-membered ring of the compound with the bridging carbon atom between C2 and C6. (*b*)–(*f*) The various arms extending from the central ring, showing the connection to the central ring.

The core of the mol­ecule represented by an eight-membered oxocine ring, which is linked in the center by a carbon atom, C1 bridging between C2 and C6, and includes an ether group as shown in Fig. 3 ▸ *a*. Bond lengths, listed in Table 1 ▸, clearly indicate the presence of a C=C double bond with a bond length of 1.357 (3) Å and also featuring a short C—C bond with a bond length of 1.494 (3) Å,. Also inter­esting to note is the stark difference between the C—O bond lengths within the ring, ranging from 1.333 (3) to 1.462 (2) Å. The presence of the double bond, the short C—C bond and the great variation within the C—O bonds is due to the five arms of the structure, which extend from the central ring as shown in Fig. 3 ▸ *b*–3*f* and are described in Table 2 ▸.

## Supra­molecular features   

**Figure 4:**
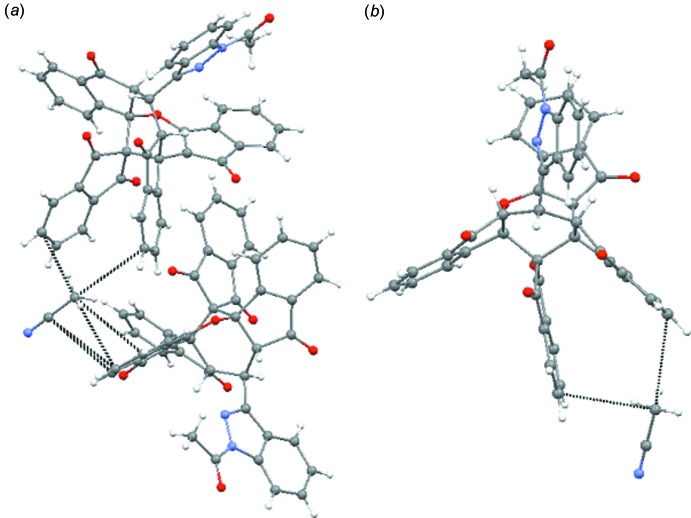
(*a*) van der Waals inter­actions between the aceto­nitrile mol­ecules and the title compound: one aceto­nitrile mol­ecule inter­acts with up to three arms. (*b*) Illustration of the rigidity that is enforced upon two arms of the title compound as a result of van der Waals inter­actions.

The packing of the crystal structure indicates that the aceto­nitrile mol­ecules inter­act with up to three different aromatic π systems belonging to arms 1–3; these inter­actions can be seen in Fig. 4 ▸ *a*. The inter­actions between aceto­nitrile and arms 2 and 3 also force some rigidity upon the structure, as seen in Fig. 4 ▸ *b.* It is worth noting that the inter­action seen in Fig. 4 ▸ *b* is not observed in the asymmetric unit, but in the extended packing of the crystal. These inter­actions are listed in Table 3 ▸.

**Figure 5:**
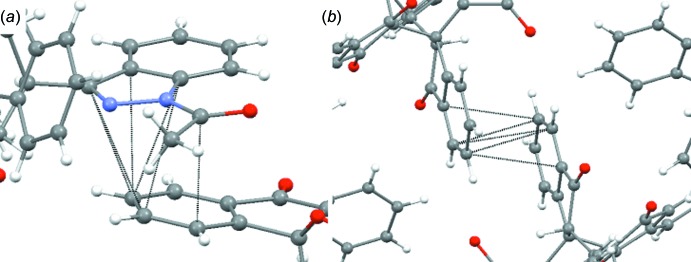
π–π stacking between (*a*) arms 4 and 5 of adjacent mol­ecules and (*b*) arms 1 and 2 of the title compound.

A second inter­action, which contributes to the crystal packing, is a π–π inter­action between arms 4 and 5, as seen in Fig. 5 ▸ *a*. A third inter­action, which contributes to the crystal packing, is a π–π inter­action between arms 1 and 2, as seen in Fig. 5 ▸ *b*. These inter­actions are listed in Table 3 ▸.

**Figure 6:**
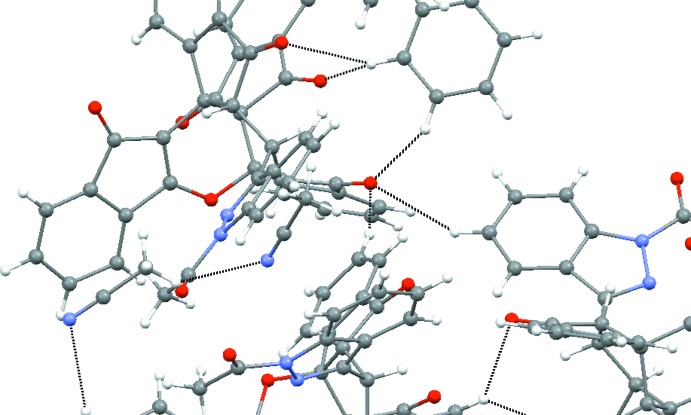
Part of the C—H⋯O=C bonding pattern between mol­ecules of the title compound and also part of the C—H⋯N≡C bonding pattern between the aceto­nitrile mol­ecules.

Finally, a hydrogen-bonding network (Table 4 ▸) is observed throughout the crystal, consisting of a C—H⋯O=C bonding pattern between the mol­ecules of the title compound, and a C—H⋯N≡C bonding pattern between the aceto­nitrile mol­ecules, as seen in Fig. 6 ▸.

## Database survey   

A search of the Cambridge Structural Database (CSD Version 5.39, update of August 2018; Groom *et al.*, 2016 ▸) for the mol­ecular formula (C_46_H_26_N_2_O_7_) and for unit-cell dimensions yielded no results. Searching for the various arms yielded 72 hits for indanone and 38 hits for indandione. Similar structures that contain eight-membered rings and are the result of aldol condensation, namely 1-(1,3-dioxoindan-2-yl­idene)-2-[spiro-1,3-indandione-2,18′-(5′*H*,9b′*H*,12′*H*,16b′*H*-5′,12′-dioxo-9b′,16b′-methano-11′-methyl­tri­indeno­(1,2-*b*:1,2-*d*:1,2-*f*)oxocin-17-yl)]inden-3-yl acetate and spiro­(1,3-indandione-2,10′-5′*H*,9b′*H*,10′*H*,16′*H*-5′,16′-dioxobenzo[*a*]di­indeno[1,2-*f*:1,2-*h*]azulen-11′-yl acetate) have been published previously (refcodes MEKQIC, MEKQEY; Jacob *et al.*, 2000 ▸).

## Synthesis and crystallization   

The synthetic procedure for the title compound will be published elsewhere. The title compound was crystallized in HPLC/gradient grade aceto­nitrile (99.9%) obtained from Sigma (CAS 75-05-8) by slow evaporation at a temperature of 277 K over the course of several weeks, resulting in yellow crystals.

## Refinement   

Crystal data, data collection and structure refinement details are summarized in Table 5 ▸. Hydrogen atoms were placed at calculated positions (C—H = 0.93–0.98 Å) and refined in riding mode, with *U*_iso_(H) = 1.2*U*_eq_(C) for CH and CH_2_ groups and 1.5*U*_eq_(C) for CH_3_ groups.

## Supplementary Material

Crystal structure: contains datablock(s) I. DOI: 10.1107/S2056989018011763/zq2241sup1.cif

Structure factors: contains datablock(s) I. DOI: 10.1107/S2056989018011763/zq2241Isup2.hkl

CCDC reference: 1858143

Additional supporting information:  crystallographic information; 3D view; checkCIF report

## Figures and Tables

**Table 1:** Bond lengths (Å) in the central eight-membered ring of the mol­ecule

Atom pair	Bond length	Atom pair	Bond length
C1—C2	1.547 (3)	C5—C6	1.554 (3)
C1—C6	1.576 (3)	C6—C7	1.494 (3)
C2—C3	1.552 (3)	C7=C8	1.357 (3)
C3—C4	1.546 (3)	O1—C8	1.332 (3)
C4—C5	1.544 (3)	O1—C2	1.462 (2)

**Table 2:** The various arms extending from the central ring

Arm designation	Carbon atoms shared with central ring	IUPAC name
1 (Fig. 3 ▸ *b*)	C5, C6	1-indanone
2 (Fig. 3 ▸ *c*)	C2, C3	1-indanone
3 (Fig. 3 ▸ *d*)	C1	1,3-indandione
4 (Fig. 3 ▸ *e*)	C4	1-(1*H*-indazol-1-yl)ethanone
5 (Fig. 3 ▸ *f*)	C7, C8	1-*H*-inden-1-one

**Table 3:** Packing inter­actions found inside the crystal lattice (Å) Shortest bond distances that were found are shown for the various inter­actions.

Inter­action	C⋯C
C12 (Arm 1)⋯C97^i^ (Aceto­nitrile)	3.489 (3)
C26 (Arm 3)⋯C97^i^ (Aceto­nitrile)	3.475 (3)
C58 (Arm 2′)⋯C97^ii^ (Aceto­nitrile)	3.317 (3)
C66 (Arm 1)⋯C66^i^ (Arm 1′)	3.388 (2)
C32 (Arm 4)⋯C89^iv^ (Arm 2′)	3.381 (3)

Symmetry codes: (i) 1 − *x*, 2 − *y*, 1 − *z*; (ii) *x* − 1, *y*, *z*; (iii) *x*, 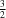 − *y*, −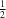 + *z*.

**Table 4:** Hydrogen-bond geometry (Å, °)

*D*—H⋯*A*	*D*—H	H⋯*A*	*D*⋯*A*	*D*—H⋯*A*
C14—H14⋯O11^i^	0.93	2.48	3.217 (2)	136
C19—H19⋯O9^ii^	0.93	2.58	3.277 (3)	132
C26—H26⋯O13	0.93	2.56	3.147 (3)	121
C39—H39*B*⋯O7^iii^	0.96	2.49	3.443 (3)	174
C44—H44⋯O2^iii^	0.93	2.59	3.446 (3)	153
C65—H65⋯O14^iv^	0.93	2.40	3.112 (3)	133
C80—H80⋯O5^i^	0.93	2.33	3.235 (2)	164
C82—H82⋯O3^v^	0.93	2.51	3.278 (3)	141
C83—H83⋯O4^v^	0.93	2.53	3.289 (2)	139
C88—H88⋯O7^i^	0.93	2.53	3.313 (3)	142
C89—H89⋯O3^vi^	0.93	2.44	3.323 (3)	159
C93—H93*B*⋯O5^vii^	0.96	2.57	3.341 (4)	137
C97—H97*B*⋯O10	0.96	2.31	3.072 (3)	1361
C51—H51⋯N3	0.98	2.55	3.000 (3)	108

Symmetry codes: (i) 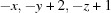; (ii) 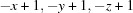; (iii) 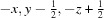; (iv) 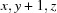; (v) 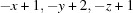; (vi) 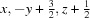; (vii) 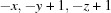.

**Table 5:** Experimental details

Crystal data	
Chemical formula	C_46_H_26_N_2_O_7_·1.5C_2_H_3_N
*M*_r_	780.27
Crystal system, space group	Monoclinic, *P*2_1_/*c*
Temperature (K)	100
*a*, *b*, *c* (Å)	13.5195 (3), 13.0697 (3), 42.9248 (9)
β (°)	92.475 (2)
*V* (Å^3^)	7577.6 (3)
*Z*	4
Radiation type	Mo *K*α
μ (mm^−1^)	0.09
Crystal size (mm)	0.32 × 0.31 × 0.29

Data collection	
Diffractometer	Rigaku Oxford Diffraction XtaLAB Synergy, Dualflex, HyPix
Absorption correction	Multi-scan (*CrysAlis PRO*; Rigaku OD, 2018 ▸)
*T*_min_, *T*_max_	0.723, 1.000
No. of measured, independent and observed [*I* > 2σ(*I*)] reflections	81627, 13290, 11196
*R*_int_	0.050
(sin θ/λ)_max_ (Å^−1^)	0.594

Refinement	
*R*[*F* ^2^ > 2σ(*F* ^2^)], *wR*(*F* ^2^), *S*	0.047, 0.122, 1.03
No. of reflections	13290
No. of parameters	1077
H-atom treatment	H-atom parameters constrained
Δρ_max_, Δρ_min_ (e Å^−3^)	1.15, −0.43

Computer programs: *CrysAlis PRO* (Rigaku OD, 2018 ▸), *SHELXT* (Sheldrick, 2015*a* ▸), *SHELXL2018* (Sheldrick, 2015*b* ▸) and *OLEX2* (Dolomanov *et al.*, 2009 ▸).

## supplementary crystallographic information

### Crystal data

**Table:**

C_46_H_26_N_2_O_7_·1.5C_2_H_3_N	*F*(000) = 3240
*M**_r_* = 780.27	*D*_x_ = 1.368 Mg m^−^^3^
Monoclinic, *P*2_1_/*c*	Mo *K*α radiation, λ = 0.71073 Å
*a* = 13.5195 (3) Å	Cell parameters from 32475 reflections
*b* = 13.0697 (3) Å	θ = 2.4–25.1°
*c* = 42.9248 (9) Å	µ = 0.09 mm^−^^1^
β = 92.475 (2)°	*T* = 100 K
*V* = 7577.6 (3) Å^3^	Cube, clear yellow
*Z* = 4	0.32 × 0.31 × 0.29 mm

### Data collection

**Table:**

Rigaku Oxford Diffraction XtaLAB Synergy, Dualflex, HyPix diffractometer	11196 reflections with *I* > 2σ(*I*)
ω scans	*R*_int_ = 0.050
Absorption correction: multi-scan (CrysAlis PRO; Rigaku OD, 2018)	θ_max_ = 25.0°, θ_min_ = 2.2°
*T*_min_ = 0.723, *T*_max_ = 1.000	*h* = −16→15
81627 measured reflections	*k* = −15→15
13290 independent reflections	*l* = −50→50

### Refinement

**Table:**

Refinement on *F*^2^	0 restraints
Least-squares matrix: full	Hydrogen site location: inferred from neighbouring sites
*R*[*F*^2^ > 2σ(*F*^2^)] = 0.047	H-atom parameters constrained
*wR*(*F*^2^) = 0.122	*w* = 1/[σ^2^(*F*_o_^2^) + (0.0537*P*)^2^ + 7.2184*P*] where *P* = (*F*_o_^2^ + 2*F*_c_^2^)/3
*S* = 1.03	(Δ/σ)_max_ = 0.001
13290 reflections	Δρ_max_ = 1.15 e Å^−^^3^
1077 parameters	Δρ_min_ = −0.43 e Å^−^^3^

### Special details

**Table:**

Geometry. All esds (except the esd in the dihedral angle between two l.s. planes) are estimated using the full covariance matrix. The cell esds are taken into account individually in the estimation of esds in distances, angles and torsion angles; correlations between esds in cell parameters are only used when they are defined by crystal symmetry. An approximate (isotropic) treatment of cell esds is used for estimating esds involving l.s. planes.
Refinement. 1. Fixed Uiso At 1.2 times of: All C(H) groups At 1.5 times of: All C(H,H,H) groups 2.a Ternary CH refined with riding coordinates: C5(H5), C51(H51), C3(H3), C4(H4), C49(H49), C50(H50) 2.b Aromatic/amide H refined with riding coordinates: C21(H21), C11(H11), C87(H87), C14(H14), C18(H18), C59(H59), C56(H56), C25(H25), C90(H90), C63(H63), C71(H71), C83(H83), C88(H88), C74(H74), C28(H28), C19(H19), C20(H20), C13(H13), C89(H89), C72(H72), C12(H12), C66(H66), C64(H64), C33(H33), C27(H27), C58(H58), C73(H73), C80(H80), C57(H57), C26(H26), C82(H82), C65(H65), C81(H81), C36(H36), C45(H45), C34(H34), C35(H35), C44(H44), C42(H42), C43(H43) 2.c Idealised Me refined as rotating group: C77(H77A,H77B,H77C), C39(H39A,H39B,H39C), C95(H95A,H95B,H95C), C97(H97A,H97B, H97C), C93(H93A,H93B,H93C)

### Fractional atomic coordinates and isotropic or equivalent isotropic displacement parameters (Å^2^)

**Table:**

	*x*	*y*	*z*	*U*_iso_*/*U*_eq_	
O4	0.28132 (9)	0.90383 (11)	0.35064 (3)	0.0193 (3)	
O5	−0.06102 (10)	0.85056 (11)	0.35809 (3)	0.0213 (3)	
O2	0.31863 (10)	1.12073 (11)	0.31418 (3)	0.0208 (3)	
O8	0.26418 (10)	0.69691 (11)	0.60733 (3)	0.0195 (3)	
O11	0.20536 (10)	0.91667 (11)	0.60793 (3)	0.0223 (3)	
O12	0.55174 (10)	0.86716 (11)	0.62155 (3)	0.0213 (3)	
O3	0.36007 (10)	0.75907 (11)	0.29532 (3)	0.0243 (3)	
O14	0.41342 (11)	0.33440 (11)	0.50365 (3)	0.0280 (3)	
O13	0.27308 (11)	0.83008 (11)	0.50520 (3)	0.0246 (3)	
O1	0.01317 (10)	0.79294 (11)	0.29860 (3)	0.0247 (3)	
O10	0.64519 (10)	0.84199 (12)	0.55745 (3)	0.0271 (3)	
O9	0.59079 (11)	0.62756 (12)	0.64619 (3)	0.0284 (3)	
O6	0.11865 (13)	0.96381 (14)	0.15127 (3)	0.0378 (4)	
N4	0.45944 (12)	0.46736 (13)	0.53442 (4)	0.0193 (4)	
N3	0.43295 (12)	0.55669 (13)	0.54928 (4)	0.0199 (4)	
N1	0.15198 (12)	0.92208 (13)	0.23177 (4)	0.0203 (4)	
N2	0.17098 (13)	0.95505 (14)	0.20192 (4)	0.0233 (4)	
O7	−0.07690 (12)	1.13677 (12)	0.28272 (4)	0.0363 (4)	
C31	0.22625 (14)	0.95317 (15)	0.24976 (4)	0.0177 (4)	
C84	0.43465 (14)	0.97866 (15)	0.64481 (4)	0.0174 (4)	
C9	0.23404 (14)	1.09819 (15)	0.31912 (4)	0.0163 (4)	
C85	0.46741 (14)	0.89491 (15)	0.62467 (4)	0.0165 (4)	
C86	0.35501 (14)	0.73340 (15)	0.65495 (4)	0.0180 (4)	
C1	0.10267 (13)	0.89504 (15)	0.33917 (4)	0.0154 (4)	
C5	0.17008 (13)	1.02282 (15)	0.29968 (4)	0.0155 (4)	
H5	0.141540	1.063153	0.282269	0.019*	
C30	0.02326 (14)	0.87827 (15)	0.36358 (4)	0.0168 (4)	
C6	0.08157 (13)	0.99648 (15)	0.32018 (4)	0.0160 (4)	
C51	0.47739 (14)	0.77743 (15)	0.56304 (4)	0.0182 (4)	
H51	0.460585	0.747922	0.542497	0.022*	
C78	0.29135 (14)	0.92053 (15)	0.61719 (4)	0.0173 (4)	
C3	0.19119 (14)	0.82563 (15)	0.29181 (4)	0.0167 (4)	
H3	0.165809	0.797237	0.271903	0.020*	
C79	0.33333 (14)	0.99391 (15)	0.64028 (5)	0.0185 (4)	
C24	0.16981 (14)	0.91786 (15)	0.39245 (4)	0.0188 (4)	
C16	0.27514 (14)	0.75419 (15)	0.30310 (4)	0.0182 (4)	
C15	0.07922 (14)	1.08829 (15)	0.34193 (4)	0.0178 (4)	
C22	0.13573 (14)	0.70507 (15)	0.32975 (4)	0.0172 (4)	
C10	0.16932 (14)	1.14016 (15)	0.34290 (4)	0.0175 (4)	
C17	0.23202 (14)	0.67764 (15)	0.32367 (4)	0.0185 (4)	
C4	0.22699 (14)	0.93492 (15)	0.28442 (4)	0.0164 (4)	
H4	0.296052	0.939779	0.292198	0.020*	
C29	0.06897 (14)	0.90144 (15)	0.39466 (5)	0.0187 (4)	
C53	0.29273 (14)	0.78625 (15)	0.55976 (4)	0.0187 (4)	
C69	0.51264 (14)	0.58956 (15)	0.56452 (4)	0.0179 (4)	
C47	0.37501 (13)	0.84789 (15)	0.60764 (4)	0.0159 (4)	
C52	0.38474 (14)	0.84001 (15)	0.57150 (4)	0.0169 (4)	
C49	0.44528 (14)	0.66568 (15)	0.61239 (4)	0.0185 (4)	
H49	0.416150	0.597643	0.609160	0.022*	
C75	0.55841 (14)	0.44390 (15)	0.54100 (4)	0.0188 (4)	
C50	0.50919 (14)	0.68575 (15)	0.58370 (4)	0.0183 (4)	
H50	0.576862	0.699402	0.591727	0.022*	
C68	0.55696 (14)	0.85675 (16)	0.55727 (4)	0.0204 (4)	
C2	0.10881 (13)	0.80699 (15)	0.31525 (4)	0.0166 (4)	
C62	0.40457 (15)	0.94379 (16)	0.55710 (4)	0.0192 (4)	
C91	0.44309 (14)	0.69583 (16)	0.66803 (5)	0.0203 (4)	
C21	0.07741 (15)	0.64118 (16)	0.34713 (5)	0.0211 (4)	
H21	0.012679	0.658879	0.351301	0.025*	
C7	−0.00955 (14)	0.97595 (17)	0.30021 (5)	0.0229 (5)	
C70	0.59492 (14)	0.52222 (15)	0.56063 (4)	0.0190 (4)	
C32	0.29768 (15)	1.00870 (16)	0.23258 (5)	0.0222 (4)	
C11	0.18869 (15)	1.21979 (16)	0.36388 (5)	0.0215 (4)	
H11	0.249531	1.253092	0.364730	0.026*	
C54	0.24333 (14)	0.72063 (15)	0.57739 (4)	0.0185 (4)	
C92	0.50673 (15)	0.65964 (16)	0.64332 (5)	0.0210 (4)	
C87	0.28013 (15)	0.76636 (15)	0.67365 (5)	0.0202 (4)	
H87	0.220249	0.790079	0.664941	0.024*	
C23	0.19807 (14)	0.90563 (14)	0.35990 (4)	0.0161 (4)	
C48	0.35832 (14)	0.73736 (15)	0.61983 (4)	0.0174 (4)	
C67	0.50352 (15)	0.95311 (16)	0.54991 (4)	0.0212 (4)	
C61	0.24575 (14)	0.78555 (15)	0.52835 (5)	0.0194 (4)	
C55	0.15761 (14)	0.67421 (15)	0.55995 (5)	0.0196 (4)	
C14	0.00486 (15)	1.11728 (16)	0.36125 (5)	0.0220 (4)	
H14	−0.055927	1.083849	0.360467	0.026*	
C60	0.15772 (14)	0.71537 (15)	0.52989 (5)	0.0201 (4)	
C18	0.27387 (15)	0.58672 (16)	0.33491 (5)	0.0231 (4)	
H18	0.338900	0.569440	0.330997	0.028*	
C76	0.38810 (15)	0.40956 (16)	0.51779 (5)	0.0219 (4)	
C59	0.08710 (15)	0.68832 (17)	0.50747 (5)	0.0240 (4)	
H59	0.087021	0.716188	0.487538	0.029*	
C56	0.08756 (15)	0.60420 (16)	0.56834 (5)	0.0241 (4)	
H56	0.087654	0.576929	0.588354	0.029*	
C25	0.22988 (15)	0.94249 (17)	0.41844 (5)	0.0236 (4)	
H25	0.297433	0.953670	0.416928	0.028*	
C90	0.46040 (16)	0.69348 (18)	0.70026 (5)	0.0258 (5)	
H90	0.520066	0.669218	0.708969	0.031*	
C46	−0.12284 (15)	0.87633 (18)	0.26946 (5)	0.0248 (5)	
C63	0.33856 (16)	1.02290 (16)	0.55081 (5)	0.0237 (4)	
H63	0.272439	1.017328	0.555708	0.028*	
C71	0.69291 (15)	0.52004 (17)	0.57239 (5)	0.0229 (4)	
H71	0.718013	0.572324	0.585122	0.027*	
C83	0.49062 (15)	1.03680 (16)	0.66609 (5)	0.0229 (4)	
H83	0.558476	1.026678	0.669028	0.027*	
C37	0.26021 (16)	1.00706 (16)	0.20159 (5)	0.0242 (4)	
C88	0.29727 (15)	0.76286 (16)	0.70568 (5)	0.0232 (4)	
H88	0.247930	0.784231	0.718652	0.028*	
C8	−0.03363 (14)	0.87959 (17)	0.29088 (5)	0.0225 (4)	
C74	0.61798 (15)	0.36241 (16)	0.53240 (5)	0.0227 (4)	
H74	0.594216	0.311555	0.518900	0.027*	
C28	0.02389 (16)	0.90879 (17)	0.42301 (5)	0.0241 (4)	
H28	−0.043755	0.898121	0.424504	0.029*	
C19	0.21529 (16)	0.52303 (16)	0.35208 (5)	0.0250 (5)	
H19	0.240940	0.461434	0.359673	0.030*	
C20	0.11859 (16)	0.55002 (16)	0.35812 (5)	0.0244 (5)	
H20	0.080612	0.506110	0.369759	0.029*	
C13	0.02341 (16)	1.19748 (16)	0.38178 (5)	0.0257 (5)	
H13	−0.025961	1.218367	0.394807	0.031*	
C89	0.38716 (16)	0.72789 (17)	0.71885 (5)	0.0264 (5)	
H89	0.397604	0.727870	0.740412	0.032*	
C72	0.75127 (15)	0.43839 (18)	0.56462 (5)	0.0262 (5)	
H72	0.816271	0.434646	0.572554	0.031*	
C12	0.11470 (16)	1.24760 (17)	0.38333 (5)	0.0260 (5)	
H12	0.125723	1.300275	0.397625	0.031*	
C66	0.53873 (16)	1.04115 (17)	0.53591 (5)	0.0266 (5)	
H66	0.604850	1.046999	0.531039	0.032*	
N5	0.41672 (18)	0.3769 (2)	0.60410 (6)	0.0511 (6)	
C64	0.37410 (17)	1.11076 (17)	0.53697 (5)	0.0297 (5)	
H64	0.331032	1.164901	0.532636	0.036*	
C33	0.38850 (16)	1.05521 (17)	0.24042 (5)	0.0276 (5)	
H33	0.413475	1.057192	0.260943	0.033*	
C27	0.08355 (17)	0.93258 (18)	0.44892 (5)	0.0294 (5)	
H27	0.055486	0.937292	0.468267	0.035*	
C58	0.01558 (15)	0.61773 (17)	0.51561 (5)	0.0270 (5)	
H58	−0.033294	0.598339	0.500876	0.032*	
C38	0.10284 (17)	0.93256 (17)	0.17729 (5)	0.0283 (5)	
C73	0.71375 (16)	0.36069 (17)	0.54484 (5)	0.0257 (5)	
H73	0.754760	0.306379	0.539972	0.031*	
C80	0.28412 (15)	1.06837 (17)	0.65691 (5)	0.0256 (5)	
H80	0.216336	1.078743	0.653858	0.031*	
N6	0.00183 (19)	0.34600 (18)	0.57506 (6)	0.0506 (6)	
C57	0.01584 (16)	0.57579 (17)	0.54527 (5)	0.0278 (5)	
H57	−0.032256	0.528031	0.549983	0.033*	
C26	0.18458 (17)	0.94969 (19)	0.44679 (5)	0.0294 (5)	
H26	0.222491	0.966241	0.464675	0.035*	
C41	−0.15187 (17)	0.97597 (17)	0.26401 (5)	0.0294 (5)	
C82	0.44174 (16)	1.11052 (18)	0.68282 (5)	0.0302 (5)	
H82	0.477272	1.150158	0.697399	0.036*	
C65	0.47261 (17)	1.11946 (18)	0.52948 (5)	0.0316 (5)	
H65	0.494293	1.178882	0.520002	0.038*	
C81	0.33991 (16)	1.12624 (18)	0.67813 (6)	0.0315 (5)	
H81	0.309056	1.176671	0.689513	0.038*	
C36	0.31232 (18)	1.05022 (18)	0.17746 (5)	0.0326 (5)	
H36	0.287906	1.048766	0.156870	0.039*	
C45	−0.17277 (18)	0.79450 (18)	0.25547 (6)	0.0344 (5)	
H45	−0.153228	0.727108	0.258981	0.041*	
C34	0.43964 (19)	1.09806 (19)	0.21652 (6)	0.0370 (6)	
H34	0.500330	1.129471	0.220980	0.044*	
C77	0.28359 (16)	0.44554 (18)	0.51910 (5)	0.0300 (5)	
H77A	0.275115	0.506815	0.506941	0.045*	
H77B	0.268680	0.459449	0.540345	0.045*	
H77C	0.239741	0.393484	0.510840	0.045*	
C40	−0.07762 (16)	1.04316 (17)	0.28265 (5)	0.0267 (5)	
C96	0.48059 (19)	0.3199 (2)	0.60569 (5)	0.0356 (6)	
C39	0.01509 (18)	0.87178 (19)	0.18588 (5)	0.0343 (5)	
H39A	−0.022888	0.910266	0.200198	0.051*	
H39B	0.036683	0.808906	0.195533	0.051*	
H39C	−0.025149	0.856933	0.167463	0.051*	
C35	0.4017 (2)	1.0950 (2)	0.18577 (6)	0.0395 (6)	
H35	0.438368	1.124442	0.170263	0.047*	
C44	−0.25598 (18)	0.8201 (2)	0.23537 (6)	0.0372 (6)	
H44	−0.293132	0.768606	0.225520	0.045*	
C42	−0.22916 (18)	0.9994 (2)	0.24452 (6)	0.0383 (6)	
H42	−0.246905	1.067116	0.240608	0.046*	
C94	0.0808 (2)	0.3293 (2)	0.58371 (6)	0.0402 (6)	
N7	0.87013 (18)	0.8519 (3)	0.47954 (6)	0.0652 (8)	
C43	−0.28084 (18)	0.9204 (2)	0.23065 (6)	0.0415 (6)	
H43	−0.335192	0.935602	0.217447	0.050*	
C98	0.86308 (18)	0.8262 (2)	0.50448 (6)	0.0433 (7)	
C95	0.5620 (2)	0.2478 (3)	0.60822 (7)	0.0524 (7)	
H95A	0.546901	0.189224	0.595387	0.079*	
H95B	0.571843	0.226514	0.629539	0.079*	
H95C	0.621213	0.279870	0.601426	0.079*	
C97	0.8546 (2)	0.7937 (3)	0.53653 (7)	0.0605 (9)	
H97A	0.828297	0.725543	0.536923	0.091*	
H97B	0.811132	0.839339	0.546894	0.091*	
H97C	0.918807	0.794790	0.546994	0.091*	
C93	0.1807 (2)	0.3063 (3)	0.59497 (9)	0.0755 (12)	
H93A	0.212121	0.263414	0.580164	0.113*	
H93B	0.178931	0.271479	0.614623	0.113*	
H93C	0.217447	0.368823	0.597584	0.113*	

### Atomic displacement parameters (Å^2^)

**Table:**

	*U*^11^	*U*^22^	*U*^33^	*U*^12^	*U*^13^	*U*^23^
O4	0.0145 (7)	0.0234 (8)	0.0200 (7)	0.0007 (6)	−0.0003 (5)	0.0000 (6)
O5	0.0159 (7)	0.0222 (8)	0.0259 (7)	−0.0003 (6)	0.0011 (6)	0.0011 (6)
O2	0.0164 (7)	0.0216 (8)	0.0244 (7)	−0.0026 (6)	0.0000 (5)	0.0014 (6)
O8	0.0182 (7)	0.0195 (7)	0.0204 (7)	−0.0034 (6)	−0.0032 (5)	0.0014 (6)
O11	0.0154 (7)	0.0240 (8)	0.0272 (7)	0.0012 (6)	−0.0030 (6)	−0.0009 (6)
O12	0.0155 (7)	0.0257 (8)	0.0226 (7)	0.0026 (6)	−0.0011 (5)	−0.0041 (6)
O3	0.0206 (8)	0.0258 (8)	0.0269 (8)	0.0052 (6)	0.0054 (6)	0.0013 (6)
O14	0.0386 (9)	0.0179 (8)	0.0270 (8)	−0.0003 (7)	−0.0052 (6)	−0.0035 (6)
O13	0.0300 (8)	0.0243 (8)	0.0193 (7)	−0.0023 (6)	−0.0008 (6)	0.0021 (6)
O1	0.0197 (7)	0.0272 (8)	0.0266 (8)	0.0002 (6)	−0.0044 (6)	−0.0044 (6)
O10	0.0193 (8)	0.0292 (9)	0.0331 (8)	−0.0027 (6)	0.0037 (6)	−0.0001 (7)
O9	0.0249 (8)	0.0348 (9)	0.0255 (8)	0.0121 (7)	0.0005 (6)	0.0029 (7)
O6	0.0477 (10)	0.0469 (11)	0.0183 (8)	0.0123 (8)	−0.0033 (7)	0.0020 (7)
N4	0.0215 (9)	0.0171 (9)	0.0193 (8)	0.0000 (7)	0.0002 (7)	−0.0032 (7)
N3	0.0213 (9)	0.0177 (9)	0.0207 (8)	0.0009 (7)	0.0005 (7)	−0.0021 (7)
N1	0.0255 (9)	0.0206 (9)	0.0146 (8)	0.0023 (7)	−0.0004 (7)	0.0008 (7)
N2	0.0305 (10)	0.0240 (10)	0.0152 (8)	0.0032 (8)	0.0005 (7)	0.0020 (7)
O7	0.0377 (9)	0.0243 (9)	0.0457 (10)	0.0009 (7)	−0.0114 (8)	0.0001 (7)
C31	0.0205 (10)	0.0151 (10)	0.0176 (10)	0.0028 (8)	0.0019 (8)	−0.0014 (8)
C84	0.0156 (9)	0.0172 (10)	0.0195 (9)	0.0002 (8)	0.0012 (7)	0.0010 (8)
C9	0.0186 (10)	0.0146 (10)	0.0154 (9)	0.0012 (8)	−0.0016 (7)	0.0032 (7)
C85	0.0169 (10)	0.0169 (10)	0.0156 (9)	0.0000 (8)	−0.0006 (7)	0.0027 (8)
C86	0.0204 (10)	0.0141 (10)	0.0194 (10)	−0.0019 (8)	−0.0002 (8)	0.0015 (8)
C1	0.0133 (9)	0.0151 (10)	0.0177 (9)	0.0012 (7)	−0.0012 (7)	0.0012 (8)
C5	0.0163 (9)	0.0154 (10)	0.0146 (9)	−0.0002 (7)	−0.0008 (7)	0.0017 (7)
C30	0.0166 (10)	0.0119 (9)	0.0219 (10)	0.0020 (7)	0.0016 (8)	0.0012 (8)
C6	0.0149 (9)	0.0152 (10)	0.0179 (9)	0.0012 (7)	−0.0010 (7)	0.0004 (8)
C51	0.0203 (10)	0.0182 (10)	0.0159 (9)	−0.0012 (8)	−0.0003 (7)	−0.0009 (8)
C78	0.0170 (10)	0.0170 (10)	0.0180 (9)	−0.0013 (8)	0.0010 (7)	0.0051 (8)
C3	0.0184 (9)	0.0167 (10)	0.0147 (9)	0.0011 (8)	−0.0010 (7)	−0.0009 (8)
C79	0.0174 (10)	0.0162 (10)	0.0219 (10)	−0.0005 (8)	−0.0001 (8)	0.0019 (8)
C24	0.0200 (10)	0.0163 (10)	0.0201 (10)	0.0024 (8)	0.0000 (8)	0.0044 (8)
C16	0.0190 (10)	0.0198 (11)	0.0157 (9)	0.0034 (8)	0.0002 (7)	−0.0035 (8)
C15	0.0212 (10)	0.0145 (10)	0.0175 (9)	0.0021 (8)	−0.0016 (8)	0.0029 (8)
C22	0.0204 (10)	0.0155 (10)	0.0155 (9)	0.0011 (8)	−0.0028 (7)	−0.0015 (8)
C10	0.0199 (10)	0.0163 (10)	0.0161 (9)	0.0030 (8)	−0.0019 (7)	0.0029 (8)
C17	0.0212 (10)	0.0177 (10)	0.0163 (9)	0.0016 (8)	−0.0014 (7)	−0.0013 (8)
C4	0.0152 (9)	0.0175 (10)	0.0165 (9)	0.0008 (8)	−0.0007 (7)	−0.0010 (8)
C29	0.0205 (10)	0.0156 (10)	0.0201 (10)	0.0009 (8)	0.0006 (8)	0.0021 (8)
C53	0.0165 (9)	0.0189 (10)	0.0202 (10)	0.0017 (8)	−0.0024 (8)	−0.0033 (8)
C69	0.0189 (10)	0.0178 (10)	0.0171 (9)	−0.0008 (8)	0.0011 (7)	0.0014 (8)
C47	0.0145 (9)	0.0156 (10)	0.0174 (9)	−0.0009 (7)	−0.0021 (7)	0.0000 (8)
C52	0.0170 (9)	0.0168 (10)	0.0167 (9)	−0.0010 (8)	−0.0017 (7)	0.0004 (8)
C49	0.0207 (10)	0.0165 (10)	0.0182 (10)	0.0019 (8)	−0.0002 (8)	0.0010 (8)
C75	0.0220 (10)	0.0188 (10)	0.0159 (9)	0.0001 (8)	0.0024 (8)	0.0017 (8)
C50	0.0173 (9)	0.0191 (10)	0.0185 (9)	0.0002 (8)	0.0000 (7)	−0.0014 (8)
C68	0.0200 (11)	0.0245 (11)	0.0166 (9)	−0.0033 (8)	0.0011 (8)	−0.0023 (8)
C2	0.0129 (9)	0.0180 (10)	0.0187 (9)	−0.0006 (8)	−0.0026 (7)	−0.0012 (8)
C62	0.0232 (10)	0.0195 (10)	0.0146 (9)	−0.0043 (8)	−0.0036 (8)	−0.0004 (8)
C91	0.0195 (10)	0.0193 (10)	0.0221 (10)	0.0012 (8)	0.0004 (8)	0.0020 (8)
C21	0.0225 (10)	0.0175 (10)	0.0236 (10)	0.0009 (8)	0.0027 (8)	−0.0016 (8)
C7	0.0112 (9)	0.0323 (12)	0.0249 (10)	0.0010 (8)	−0.0018 (8)	0.0147 (9)
C70	0.0212 (10)	0.0192 (10)	0.0169 (9)	0.0003 (8)	0.0031 (8)	0.0014 (8)
C32	0.0289 (11)	0.0164 (10)	0.0219 (10)	0.0005 (9)	0.0071 (8)	0.0008 (8)
C11	0.0251 (11)	0.0182 (11)	0.0210 (10)	−0.0020 (8)	−0.0025 (8)	0.0002 (8)
C54	0.0180 (10)	0.0173 (10)	0.0201 (10)	0.0011 (8)	−0.0009 (8)	−0.0025 (8)
C92	0.0216 (11)	0.0195 (11)	0.0217 (10)	0.0038 (8)	−0.0001 (8)	0.0031 (8)
C87	0.0197 (10)	0.0170 (10)	0.0239 (10)	0.0005 (8)	0.0015 (8)	0.0033 (8)
C23	0.0172 (10)	0.0118 (9)	0.0190 (9)	0.0009 (7)	−0.0012 (8)	0.0020 (7)
C48	0.0156 (9)	0.0159 (10)	0.0207 (10)	−0.0014 (8)	−0.0009 (7)	0.0001 (8)
C67	0.0231 (10)	0.0218 (11)	0.0182 (10)	−0.0048 (8)	−0.0039 (8)	−0.0011 (8)
C61	0.0199 (10)	0.0175 (10)	0.0206 (10)	0.0026 (8)	−0.0012 (8)	−0.0015 (8)
C55	0.0159 (9)	0.0177 (10)	0.0249 (10)	0.0024 (8)	−0.0018 (8)	−0.0053 (8)
C14	0.0211 (10)	0.0180 (10)	0.0273 (11)	0.0021 (8)	0.0047 (8)	0.0022 (8)
C60	0.0198 (10)	0.0168 (10)	0.0238 (10)	0.0025 (8)	0.0019 (8)	−0.0034 (8)
C18	0.0244 (11)	0.0212 (11)	0.0233 (10)	0.0066 (9)	−0.0013 (8)	−0.0006 (8)
C76	0.0292 (11)	0.0173 (11)	0.0188 (10)	−0.0038 (9)	−0.0035 (8)	0.0039 (8)
C59	0.0251 (11)	0.0239 (11)	0.0227 (10)	0.0044 (9)	−0.0020 (8)	−0.0028 (9)
C56	0.0263 (11)	0.0191 (11)	0.0269 (11)	0.0003 (9)	0.0008 (8)	0.0007 (9)
C25	0.0213 (10)	0.0285 (12)	0.0206 (10)	0.0011 (9)	−0.0027 (8)	0.0031 (9)
C90	0.0245 (11)	0.0319 (12)	0.0206 (10)	0.0042 (9)	−0.0019 (8)	0.0029 (9)
C46	0.0162 (10)	0.0376 (13)	0.0203 (10)	−0.0016 (9)	−0.0031 (8)	0.0033 (9)
C63	0.0239 (11)	0.0221 (11)	0.0246 (11)	−0.0008 (9)	−0.0045 (8)	0.0022 (9)
C71	0.0229 (10)	0.0259 (11)	0.0198 (10)	0.0002 (9)	0.0004 (8)	−0.0035 (8)
C83	0.0173 (10)	0.0253 (11)	0.0259 (11)	−0.0006 (8)	0.0001 (8)	−0.0043 (9)
C37	0.0318 (11)	0.0201 (11)	0.0208 (10)	0.0038 (9)	0.0036 (8)	0.0004 (8)
C88	0.0270 (11)	0.0205 (11)	0.0228 (10)	−0.0001 (9)	0.0075 (8)	0.0010 (8)
C8	0.0169 (10)	0.0317 (12)	0.0189 (10)	−0.0004 (9)	0.0001 (8)	0.0042 (9)
C74	0.0284 (11)	0.0190 (11)	0.0211 (10)	0.0002 (9)	0.0048 (8)	−0.0008 (8)
C28	0.0231 (10)	0.0276 (12)	0.0221 (10)	−0.0020 (9)	0.0051 (8)	0.0028 (9)
C19	0.0334 (12)	0.0182 (11)	0.0231 (10)	0.0069 (9)	−0.0028 (9)	0.0030 (8)
C20	0.0322 (12)	0.0185 (11)	0.0226 (10)	−0.0020 (9)	0.0015 (9)	0.0016 (8)
C13	0.0338 (12)	0.0207 (11)	0.0232 (10)	0.0038 (9)	0.0085 (9)	−0.0017 (9)
C89	0.0321 (12)	0.0295 (12)	0.0175 (10)	−0.0007 (9)	0.0003 (9)	0.0019 (9)
C72	0.0202 (10)	0.0319 (12)	0.0265 (11)	0.0050 (9)	0.0004 (8)	0.0003 (9)
C12	0.0381 (12)	0.0200 (11)	0.0198 (10)	0.0010 (9)	0.0007 (9)	−0.0050 (8)
C66	0.0259 (11)	0.0273 (12)	0.0260 (11)	−0.0102 (9)	−0.0052 (9)	0.0046 (9)
N5	0.0479 (14)	0.0571 (16)	0.0494 (14)	0.0082 (13)	0.0142 (11)	0.0171 (12)
C64	0.0328 (12)	0.0214 (12)	0.0342 (12)	−0.0021 (9)	−0.0079 (10)	0.0056 (9)
C33	0.0304 (12)	0.0240 (12)	0.0286 (11)	−0.0059 (9)	0.0053 (9)	−0.0018 (9)
C27	0.0337 (12)	0.0372 (13)	0.0178 (10)	0.0011 (10)	0.0058 (9)	0.0034 (9)
C58	0.0232 (11)	0.0250 (12)	0.0321 (12)	−0.0005 (9)	−0.0080 (9)	−0.0082 (9)
C38	0.0377 (13)	0.0269 (12)	0.0196 (11)	0.0108 (10)	−0.0058 (9)	−0.0020 (9)
C73	0.0254 (11)	0.0247 (12)	0.0277 (11)	0.0064 (9)	0.0078 (9)	0.0006 (9)
C80	0.0186 (10)	0.0230 (11)	0.0353 (12)	0.0034 (8)	0.0009 (9)	−0.0038 (9)
N6	0.0529 (15)	0.0372 (13)	0.0619 (15)	0.0033 (11)	0.0037 (12)	0.0124 (12)
C57	0.0229 (11)	0.0203 (11)	0.0402 (13)	−0.0062 (9)	0.0006 (9)	−0.0022 (10)
C26	0.0328 (12)	0.0389 (14)	0.0159 (10)	0.0006 (10)	−0.0042 (9)	0.0018 (9)
C41	0.0399 (13)	0.0242 (12)	0.0242 (11)	−0.0034 (10)	0.0013 (9)	−0.0009 (9)
C82	0.0243 (11)	0.0296 (13)	0.0365 (12)	−0.0023 (9)	−0.0003 (9)	−0.0141 (10)
C65	0.0368 (13)	0.0233 (12)	0.0337 (12)	−0.0101 (10)	−0.0083 (10)	0.0096 (10)
C81	0.0245 (11)	0.0271 (12)	0.0431 (13)	0.0023 (9)	0.0044 (10)	−0.0150 (10)
C36	0.0471 (14)	0.0279 (13)	0.0234 (11)	0.0031 (11)	0.0089 (10)	0.0053 (9)
C45	0.0446 (14)	0.0209 (12)	0.0385 (13)	0.0081 (10)	0.0106 (11)	0.0030 (10)
C34	0.0397 (14)	0.0330 (14)	0.0391 (14)	−0.0122 (11)	0.0130 (11)	−0.0002 (11)
C77	0.0263 (11)	0.0269 (12)	0.0363 (12)	−0.0042 (9)	−0.0056 (9)	−0.0015 (10)
C40	0.0280 (11)	0.0235 (12)	0.0284 (11)	0.0001 (9)	−0.0022 (9)	−0.0012 (9)
C96	0.0395 (14)	0.0432 (15)	0.0245 (12)	−0.0004 (12)	0.0052 (10)	0.0067 (11)
C39	0.0418 (14)	0.0322 (13)	0.0277 (12)	0.0003 (11)	−0.0141 (10)	−0.0011 (10)
C35	0.0517 (16)	0.0329 (14)	0.0355 (13)	−0.0064 (12)	0.0202 (12)	0.0068 (11)
C44	0.0401 (14)	0.0368 (14)	0.0341 (13)	−0.0156 (11)	−0.0074 (10)	−0.0116 (11)
C42	0.0382 (14)	0.0324 (14)	0.0436 (14)	0.0042 (11)	−0.0068 (11)	0.0035 (11)
C94	0.0470 (16)	0.0321 (14)	0.0419 (14)	−0.0078 (12)	0.0058 (12)	0.0084 (11)
N7	0.0392 (13)	0.109 (2)	0.0481 (15)	0.0146 (14)	0.0087 (11)	0.0306 (16)
C43	0.0297 (13)	0.0428 (16)	0.0504 (15)	0.0011 (11)	−0.0152 (11)	0.0120 (12)
C98	0.0276 (13)	0.0573 (18)	0.0454 (16)	0.0122 (12)	0.0071 (11)	0.0108 (13)
C95	0.0523 (17)	0.062 (2)	0.0424 (15)	0.0185 (15)	0.0022 (13)	0.0110 (14)
C97	0.071 (2)	0.063 (2)	0.0493 (17)	0.0325 (17)	0.0279 (15)	0.0133 (15)
C93	0.0429 (17)	0.099 (3)	0.084 (2)	−0.0192 (18)	−0.0034 (16)	0.043 (2)

### Geometric parameters (Å, º)

**Table:**

O4—C23	1.210 (2)	C87—C88	1.385 (3)
O5—C30	1.209 (2)	C67—C66	1.391 (3)
O2—C9	1.209 (2)	C61—C60	1.506 (3)
O8—C54	1.340 (2)	C55—C60	1.398 (3)
O8—C48	1.459 (2)	C55—C56	1.376 (3)
O11—C78	1.213 (2)	C14—H14	0.9300
O12—C85	1.209 (2)	C14—C13	1.386 (3)
O3—C16	1.211 (2)	C60—C59	1.372 (3)
O14—C76	1.212 (3)	C18—H18	0.9300
O13—C61	1.222 (2)	C18—C19	1.383 (3)
O1—C2	1.462 (2)	C76—C77	1.492 (3)
O1—C8	1.332 (3)	C59—H59	0.9300
O10—C68	1.208 (2)	C59—C58	1.392 (3)
O9—C92	1.213 (2)	C56—H56	0.9300
O6—C38	1.217 (3)	C56—C57	1.406 (3)
N4—N3	1.385 (2)	C25—H25	0.9300
N4—C75	1.390 (3)	C25—C26	1.389 (3)
N4—C76	1.397 (3)	C90—H90	0.9300
N3—C69	1.309 (3)	C90—C89	1.374 (3)
N1—N2	1.386 (2)	C46—C8	1.485 (3)
N1—C31	1.305 (3)	C46—C41	1.377 (3)
N2—C37	1.385 (3)	C46—C45	1.387 (3)
N2—C38	1.403 (3)	C63—H63	0.9300
O7—C40	1.223 (3)	C63—C64	1.388 (3)
C31—C4	1.506 (3)	C71—H71	0.9300
C31—C32	1.437 (3)	C71—C72	1.377 (3)
C84—C85	1.475 (3)	C83—H83	0.9300
C84—C79	1.389 (3)	C83—C82	1.386 (3)
C84—C83	1.387 (3)	C37—C36	1.397 (3)
C9—C5	1.534 (3)	C88—H88	0.9300
C9—C10	1.478 (3)	C88—C89	1.395 (3)
C85—C47	1.547 (3)	C74—H74	0.9300
C86—C91	1.384 (3)	C74—C73	1.379 (3)
C86—C87	1.387 (3)	C28—H28	0.9300
C86—C48	1.511 (3)	C28—C27	1.381 (3)
C1—C30	1.548 (3)	C19—H19	0.9300
C1—C6	1.576 (3)	C19—C20	1.389 (3)
C1—C2	1.547 (3)	C20—H20	0.9300
C1—C23	1.541 (2)	C13—H13	0.9300
C5—H5	0.9800	C13—C12	1.396 (3)
C5—C6	1.554 (3)	C89—H89	0.9300
C5—C4	1.544 (3)	C72—H72	0.9300
C30—C29	1.478 (3)	C72—C73	1.404 (3)
C6—C15	1.522 (3)	C12—H12	0.9300
C6—C7	1.494 (3)	C66—H66	0.9300
C51—H51	0.9800	C66—C65	1.379 (3)
C51—C52	1.552 (3)	N5—C96	1.140 (3)
C51—C50	1.541 (3)	C64—H64	0.9300
C51—C68	1.522 (3)	C64—C65	1.388 (3)
C78—C79	1.475 (3)	C33—H33	0.9300
C78—C47	1.546 (3)	C33—C34	1.380 (3)
C3—H3	0.9800	C27—H27	0.9300
C3—C16	1.532 (3)	C27—C26	1.391 (3)
C3—C4	1.546 (3)	C58—H58	0.9300
C3—C2	1.552 (3)	C58—C57	1.386 (3)
C79—C80	1.393 (3)	C38—C39	1.487 (3)
C24—C29	1.387 (3)	C73—H73	0.9300
C24—C23	1.473 (3)	C80—H80	0.9300
C24—C25	1.389 (3)	C80—C81	1.382 (3)
C16—C17	1.471 (3)	N6—C94	1.137 (3)
C15—C10	1.393 (3)	C57—H57	0.9300
C15—C14	1.383 (3)	C26—H26	0.9300
C22—C17	1.386 (3)	C41—C40	1.533 (3)
C22—C2	1.508 (3)	C41—C42	1.345 (3)
C22—C21	1.388 (3)	C82—H82	0.9300
C10—C11	1.394 (3)	C82—C81	1.398 (3)
C17—C18	1.393 (3)	C65—H65	0.9300
C4—H4	0.9800	C81—H81	0.9300
C29—C28	1.388 (3)	C36—H36	0.9300
C53—C52	1.497 (3)	C36—C35	1.376 (4)
C53—C54	1.341 (3)	C45—H45	0.9300
C53—C61	1.465 (3)	C45—C44	1.427 (3)
C69—C50	1.504 (3)	C34—H34	0.9300
C69—C70	1.434 (3)	C34—C35	1.396 (4)
C47—C52	1.566 (3)	C77—H77A	0.9600
C47—C48	1.556 (3)	C77—H77B	0.9600
C52—C62	1.519 (3)	C77—H77C	0.9600
C49—H49	0.9800	C96—C95	1.450 (4)
C49—C50	1.557 (3)	C39—H39A	0.9600
C49—C92	1.537 (3)	C39—H39B	0.9600
C49—C48	1.547 (3)	C39—H39C	0.9600
C75—C70	1.402 (3)	C35—H35	0.9300
C75—C74	1.395 (3)	C44—H44	0.9300
C50—H50	0.9800	C44—C43	1.367 (4)
C68—C67	1.479 (3)	C42—H42	0.9300
C62—C67	1.391 (3)	C42—C43	1.369 (4)
C62—C63	1.385 (3)	C94—C93	1.446 (4)
C91—C92	1.472 (3)	N7—C98	1.130 (3)
C91—C90	1.393 (3)	C43—H43	0.9300
C21—H21	0.9300	C98—C97	1.449 (4)
C21—C20	1.389 (3)	C95—H95A	0.9600
C7—C8	1.357 (3)	C95—H95B	0.9600
C7—C40	1.458 (3)	C95—H95C	0.9600
C70—C71	1.397 (3)	C97—H97A	0.9600
C32—C37	1.403 (3)	C97—H97B	0.9600
C32—C33	1.398 (3)	C97—H97C	0.9600
C11—H11	0.9300	C93—H93A	0.9600
C11—C12	1.379 (3)	C93—H93B	0.9600
C54—C55	1.482 (3)	C93—H93C	0.9600
C87—H87	0.9300		
			
C56···C97	3.381		
			
C54—O8—C48	114.40 (15)	O13—C61—C53	127.64 (19)
C8—O1—C2	114.55 (16)	O13—C61—C60	126.34 (18)
N3—N4—C75	110.99 (15)	C53—C61—C60	105.99 (17)
N3—N4—C76	120.31 (16)	C60—C55—C54	106.07 (17)
C75—N4—C76	128.46 (17)	C56—C55—C54	132.23 (19)
C69—N3—N4	106.46 (16)	C56—C55—C60	121.70 (18)
C31—N1—N2	106.35 (16)	C15—C14—H14	120.9
N1—N2—C38	119.31 (17)	C15—C14—C13	118.17 (19)
C37—N2—N1	111.01 (16)	C13—C14—H14	120.9
C37—N2—C38	129.68 (18)	C55—C60—C61	107.87 (17)
N1—C31—C4	120.49 (17)	C59—C60—C61	130.80 (19)
N1—C31—C32	111.75 (17)	C59—C60—C55	121.31 (19)
C32—C31—C4	127.65 (18)	C17—C18—H18	121.2
C79—C84—C85	110.10 (17)	C19—C18—C17	117.67 (19)
C83—C84—C85	128.56 (18)	C19—C18—H18	121.2
C83—C84—C79	121.33 (18)	O14—C76—N4	119.38 (19)
O2—C9—C5	125.23 (17)	O14—C76—C77	124.24 (19)
O2—C9—C10	128.56 (18)	N4—C76—C77	116.38 (18)
C10—C9—C5	106.10 (15)	C60—C59—H59	121.2
O12—C85—C84	126.58 (18)	C60—C59—C58	117.7 (2)
O12—C85—C47	125.06 (17)	C58—C59—H59	121.2
C84—C85—C47	108.36 (15)	C55—C56—H56	121.5
C91—C86—C87	120.80 (18)	C55—C56—C57	116.9 (2)
C91—C86—C48	110.78 (17)	C57—C56—H56	121.5
C87—C86—C48	128.33 (18)	C24—C25—H25	121.5
C30—C1—C6	110.73 (15)	C26—C25—C24	117.05 (19)
C2—C1—C30	113.74 (15)	C26—C25—H25	121.5
C2—C1—C6	107.22 (15)	C91—C90—H90	120.8
C23—C1—C30	102.10 (15)	C89—C90—C91	118.37 (19)
C23—C1—C6	110.57 (15)	C89—C90—H90	120.8
C23—C1—C2	112.49 (15)	C41—C46—C8	107.21 (19)
C9—C5—H5	105.3	C41—C46—C45	121.7 (2)
C9—C5—C6	105.32 (15)	C45—C46—C8	131.1 (2)
C9—C5—C4	115.41 (15)	C62—C63—H63	121.0
C6—C5—H5	105.3	C62—C63—C64	118.0 (2)
C4—C5—H5	105.3	C64—C63—H63	121.0
C4—C5—C6	119.03 (16)	C70—C71—H71	120.9
O5—C30—C1	125.87 (17)	C72—C71—C70	118.2 (2)
O5—C30—C29	126.24 (18)	C72—C71—H71	120.9
C29—C30—C1	107.88 (15)	C84—C83—H83	121.2
C5—C6—C1	110.65 (15)	C82—C83—C84	117.58 (19)
C15—C6—C1	110.73 (15)	C82—C83—H83	121.2
C15—C6—C5	102.11 (15)	N2—C37—C32	106.05 (17)
C7—C6—C1	105.78 (15)	N2—C37—C36	132.3 (2)
C7—C6—C5	110.55 (15)	C36—C37—C32	121.6 (2)
C7—C6—C15	117.06 (16)	C87—C88—H88	119.4
C52—C51—H51	105.1	C87—C88—C89	121.24 (19)
C50—C51—H51	105.1	C89—C88—H88	119.4
C50—C51—C52	118.88 (16)	O1—C8—C7	127.61 (18)
C68—C51—H51	105.1	O1—C8—C46	119.59 (19)
C68—C51—C52	105.21 (16)	C7—C8—C46	112.79 (19)
C68—C51—C50	116.21 (16)	C75—C74—H74	121.5
O11—C78—C79	125.63 (18)	C73—C74—C75	117.0 (2)
O11—C78—C47	126.11 (18)	C73—C74—H74	121.5
C79—C78—C47	108.23 (15)	C29—C28—H28	121.4
C16—C3—H3	105.8	C27—C28—C29	117.29 (19)
C16—C3—C4	113.20 (15)	C27—C28—H28	121.4
C16—C3—C2	103.99 (15)	C18—C19—H19	119.6
C4—C3—H3	105.8	C18—C19—C20	120.77 (19)
C4—C3—C2	121.20 (16)	C20—C19—H19	119.6
C2—C3—H3	105.8	C21—C20—C19	121.4 (2)
C84—C79—C78	110.44 (17)	C21—C20—H20	119.3
C84—C79—C80	121.22 (19)	C19—C20—H20	119.3
C80—C79—C78	128.32 (18)	C14—C13—H13	119.3
C29—C24—C23	110.13 (17)	C14—C13—C12	121.37 (19)
C29—C24—C25	121.53 (18)	C12—C13—H13	119.3
C25—C24—C23	128.34 (18)	C90—C89—C88	120.58 (19)
O3—C16—C3	125.36 (18)	C90—C89—H89	119.7
O3—C16—C17	127.32 (18)	C88—C89—H89	119.7
C17—C16—C3	107.28 (16)	C71—C72—H72	119.6
C10—C15—C6	111.13 (16)	C71—C72—C73	120.74 (19)
C14—C15—C6	128.23 (18)	C73—C72—H72	119.6
C14—C15—C10	120.41 (18)	C11—C12—C13	120.68 (19)
C17—C22—C2	111.26 (17)	C11—C12—H12	119.7
C17—C22—C21	120.37 (18)	C13—C12—H12	119.7
C21—C22—C2	128.36 (18)	C67—C66—H66	121.0
C15—C10—C9	109.98 (17)	C65—C66—C67	118.0 (2)
C15—C10—C11	121.48 (18)	C65—C66—H66	121.0
C11—C10—C9	128.53 (18)	C63—C64—H64	119.3
C22—C17—C16	109.69 (17)	C65—C64—C63	121.3 (2)
C22—C17—C18	121.74 (19)	C65—C64—H64	119.3
C18—C17—C16	128.48 (18)	C32—C33—H33	121.3
C31—C4—C5	108.61 (15)	C34—C33—C32	117.4 (2)
C31—C4—C3	111.12 (16)	C34—C33—H33	121.3
C31—C4—H4	107.0	C28—C27—H27	119.2
C5—C4—C3	115.73 (15)	C28—C27—C26	121.64 (19)
C5—C4—H4	107.0	C26—C27—H27	119.2
C3—C4—H4	107.0	C59—C58—H58	119.4
C24—C29—C30	110.10 (17)	C57—C58—C59	121.23 (19)
C24—C29—C28	121.29 (19)	C57—C58—H58	119.4
C28—C29—C30	128.60 (18)	O6—C38—N2	119.1 (2)
C54—C53—C52	122.47 (17)	O6—C38—C39	125.5 (2)
C54—C53—C61	107.98 (17)	N2—C38—C39	115.44 (18)
C61—C53—C52	129.32 (18)	C74—C73—C72	122.1 (2)
N3—C69—C50	120.24 (17)	C74—C73—H73	119.0
N3—C69—C70	111.48 (17)	C72—C73—H73	119.0
C70—C69—C50	128.27 (17)	C79—C80—H80	121.3
C85—C47—C52	113.18 (15)	C81—C80—C79	117.44 (19)
C85—C47—C48	109.61 (15)	C81—C80—H80	121.3
C78—C47—C85	102.46 (15)	C56—C57—H57	119.4
C78—C47—C52	113.40 (15)	C58—C57—C56	121.1 (2)
C78—C47—C48	111.25 (15)	C58—C57—H57	119.4
C48—C47—C52	106.96 (15)	C25—C26—C27	121.2 (2)
C51—C52—C47	111.70 (15)	C25—C26—H26	119.4
C53—C52—C51	109.94 (16)	C27—C26—H26	119.4
C53—C52—C47	105.09 (15)	C46—C41—C40	106.12 (19)
C53—C52—C62	116.14 (16)	C42—C41—C46	121.9 (2)
C62—C52—C51	102.52 (15)	C42—C41—C40	131.9 (2)
C62—C52—C47	111.61 (16)	C83—C82—H82	119.5
C50—C49—H49	105.9	C83—C82—C81	121.1 (2)
C92—C49—H49	105.9	C81—C82—H82	119.5
C92—C49—C50	113.21 (16)	C66—C65—C64	120.9 (2)
C92—C49—C48	103.88 (15)	C66—C65—H65	119.6
C48—C49—H49	105.9	C64—C65—H65	119.6
C48—C49—C50	120.92 (16)	C80—C81—C82	121.4 (2)
N4—C75—C70	105.78 (17)	C80—C81—H81	119.3
N4—C75—C74	132.58 (19)	C82—C81—H81	119.3
C74—C75—C70	121.62 (19)	C37—C36—H36	121.8
C51—C50—C49	115.83 (16)	C35—C36—C37	116.4 (2)
C51—C50—H50	107.1	C35—C36—H36	121.8
C69—C50—C51	110.46 (15)	C46—C45—H45	122.0
C69—C50—C49	108.86 (16)	C46—C45—C44	115.9 (2)
C69—C50—H50	107.1	C44—C45—H45	122.0
C49—C50—H50	107.1	C33—C34—H34	119.5
O10—C68—C51	126.50 (19)	C33—C34—C35	121.1 (2)
O10—C68—C67	127.61 (19)	C35—C34—H34	119.5
C67—C68—C51	105.82 (16)	C76—C77—H77A	109.5
O1—C2—C1	110.26 (15)	C76—C77—H77B	109.5
O1—C2—C3	110.31 (15)	C76—C77—H77C	109.5
O1—C2—C22	106.47 (15)	H77A—C77—H77B	109.5
C1—C2—C3	111.97 (15)	H77A—C77—H77C	109.5
C22—C2—C1	113.71 (15)	H77B—C77—H77C	109.5
C22—C2—C3	103.82 (15)	O7—C40—C7	126.6 (2)
C67—C62—C52	110.90 (17)	O7—C40—C41	125.4 (2)
C63—C62—C52	128.49 (18)	C7—C40—C41	108.01 (19)
C63—C62—C67	120.61 (19)	N5—C96—C95	179.1 (3)
C86—C91—C92	109.94 (17)	C38—C39—H39A	109.5
C86—C91—C90	121.00 (19)	C38—C39—H39B	109.5
C90—C91—C92	129.03 (18)	C38—C39—H39C	109.5
C22—C21—H21	121.0	H39A—C39—H39B	109.5
C22—C21—C20	118.03 (19)	H39A—C39—H39C	109.5
C20—C21—H21	121.0	H39B—C39—H39C	109.5
C8—C7—C6	121.15 (18)	C36—C35—C34	122.7 (2)
C8—C7—C40	105.57 (18)	C36—C35—H35	118.7
C40—C7—C6	132.4 (2)	C34—C35—H35	118.7
C75—C70—C69	105.28 (17)	C45—C44—H44	120.1
C71—C70—C69	134.30 (19)	C43—C44—C45	119.8 (2)
C71—C70—C75	120.39 (19)	C43—C44—H44	120.1
C37—C32—C31	104.82 (18)	C41—C42—H42	121.1
C33—C32—C31	134.31 (19)	C41—C42—C43	117.8 (2)
C33—C32—C37	120.83 (19)	C43—C42—H42	121.1
C10—C11—H11	121.1	N6—C94—C93	179.0 (3)
C12—C11—C10	117.85 (19)	C44—C43—C42	122.8 (2)
C12—C11—H11	121.1	C44—C43—H43	118.6
O8—C54—C53	126.87 (18)	C42—C43—H43	118.6
O8—C54—C55	121.15 (17)	N7—C98—C97	179.6 (4)
C53—C54—C55	111.98 (17)	C96—C95—H95A	109.5
O9—C92—C49	125.08 (18)	C96—C95—H95B	109.5
O9—C92—C91	127.74 (18)	C96—C95—H95C	109.5
C91—C92—C49	107.14 (16)	H95A—C95—H95B	109.5
C86—C87—H87	121.0	H95A—C95—H95C	109.5
C88—C87—C86	117.96 (18)	H95B—C95—H95C	109.5
C88—C87—H87	121.0	C98—C97—H97A	109.5
O4—C23—C1	125.15 (17)	C98—C97—H97B	109.5
O4—C23—C24	126.65 (17)	C98—C97—H97C	109.5
C24—C23—C1	108.20 (15)	H97A—C97—H97B	109.5
O8—C48—C86	106.96 (15)	H97A—C97—H97C	109.5
O8—C48—C47	110.46 (15)	H97B—C97—H97C	109.5
O8—C48—C49	111.29 (15)	C94—C93—H93A	109.5
C86—C48—C47	112.23 (16)	C94—C93—H93B	109.5
C86—C48—C49	103.93 (15)	C94—C93—H93C	109.5
C49—C48—C47	111.72 (15)	H93A—C93—H93B	109.5
C62—C67—C68	110.09 (18)	H93A—C93—H93C	109.5
C62—C67—C66	121.2 (2)	H93B—C93—H93C	109.5
C66—C67—C68	128.56 (19)		

### Hydrogen-bond geometry (Å, º)

**Table:**

*D*—H···*A*	*D*—H	H···*A*	*D*···*A*	*D*—H···*A*
C14—H14···O11^i^	0.93	2.48	3.217 (2)	136
C19—H19···O9^ii^	0.93	2.58	3.277 (3)	132
C26—H26···O13	0.93	2.56	3.147 (3)	121
C39—H39*B*···O7^iii^	0.96	2.49	3.443 (3)	174
C44—H44···O2^iii^	0.93	2.59	3.446 (3)	153
C65—H65···O14^iv^	0.93	2.40	3.112 (3)	133
C80—H80···O5^i^	0.93	2.33	3.235 (2)	164
C82—H82···O3^v^	0.93	2.51	3.278 (3)	141
C83—H83···O4^v^	0.93	2.53	3.289 (2)	139
C88—H88···O7^i^	0.93	2.53	3.313 (3)	142
C89—H89···O3^vi^	0.93	2.44	3.323 (3)	159
C93—H93*B*···O5^vii^	0.96	2.57	3.341 (4)	137
C97—H97*B*···O10	0.96	2.31	3.072 (3)	1361
C51—H51···N3	0.98	2.55	3.000 (3)	108

Symmetry codes: (i) −*x*, −*y*+2, −*z*+1; (ii) −*x*+1, −*y*+1, −*z*+1; (iii) −*x*, *y*−1/2, −*z*+1/2; (iv) *x*, *y*+1, *z*; (v) −*x*+1, −*y*+2, −*z*+1; (vi) *x*, −*y*+3/2, *z*+1/2; (vii) −*x*, −*y*+1, −*z*+1.
